# Development of the Prevention of Suicide Behaviour in Prisons: Enhancing Access to Therapy (PROSPECT) logic model and implementation strategies

**DOI:** 10.1192/bjb.2024.22

**Published:** 2024-10

**Authors:** Rebecca Crook, Charlotte Lennox, Yvonne Awenat, Dawn Edge, Sarah Knowles, David Honeywell, Patricia Gooding, Gillian Haddock, Helen Brooks, Daniel Pratt

**Affiliations:** 1 University of Manchester, Manchester, UK; 2 Manchester Academic Health Science Centre, Manchester, UK; 3 Greater Manchester NHS Foundation Trust, Manchester, UK; 4 University of York, York, UK; 5 Arden University, Coventry, UK

**Keywords:** Implementation, logic model, suicide, prisoners, psychological intervention

## Abstract

**Aims and method:**

This study aimed to develop and articulate a logic model and programme theories for implementing a new cognitive–behavioural suicide prevention intervention for men in prison who are perceived to be at risk of death by suicide. Semi-structured one-to-one interviews with key stakeholders and a combination of qualitative analysis techniques were used to develop programme theories.

**Results:**

Interviews with 28 stakeholders resulted in five programme theories, focusing on: trust, willingness and engagement; readiness and ability; assessment and formulation; practitioner delivering the ‘change work’ stage of the intervention face-to-face in a prison environment; and practitioner training, integrating the intervention and onward care. Each theory provides details of what contextual factors need to be considered at each stage, and what activities can facilitate achieving the intended outcomes of the intervention, both intermediate and long term.

**Clinical implications:**

The PROSPECT implementation strategy developed from the five theories can be adapted to different situations and environments.

Understanding how complex interventions work and how their casual pathways interact with implementation strategies requires careful consideration. The Medical Research Council (MRC) guidance for evaluating complex interventions has recently been updated and now recognises the importance of understanding that interventions and implementation strategies must consider their context and wider systems, in addition to measuring effectiveness.^1^

Logic models diagrammatically illustrate the causal processes between core components and intended outcomes of an intervention. They are also beneficial in understanding the dynamic implementation process, where implementation may occur across multiple levels within a number of organisations,^2,3^ and provide a consistent and structured approach to implementation.^4^ Mills and colleagues^4^ have provided a typology of logic models, consisting of four types that vary according to whether or not the model describes the relationships between factors (e.g. between activities and outcomes) and whether or not the model considers context (e.g. individual or environmental characteristics that can affect, or be affected by, implementation). A type 4 logic model dubbed the ‘real-world’ logic model (RWLM)^5^ includes both the causal relationships between model factors *and* the context of the intervention. This type of logic model captures how an intervention's success is dependent on its ability to adapt to different situations and environments. In the development of the RWLM, aspects of implementation science were included, with the introduction of the Promoting Action on Research Implementation in Health Services (PARIHS) framework. This framework conceptualises successful implementation as a function of the nature and type of evidence, the context of the environment where the intervention will be implemented and how implementation is facilitated.^6^

## Prevention of Suicide Behaviour in Prisons: Enhancing Access to Therapy (PROSPECT)

The PROSPECT programme aims to refine and evaluate a psychological intervention, cognitive–behavioural suicide prevention (CBSP),^7^ for men in prison who are identified as being at risk of death by suicide, through a series of interrelated work packages. The CBSP intervention comprises a formulation-driven approach to understanding suicide schema and a tailored intervention that is delivered over a 6-month period of up to 20 one-to-one talking therapy sessions with a CBSP-trained practitioner.^7^

Prior to being evaluated in a randomised controlled trial (RCT) and concurrent process evaluation, CBSP, as applied to prison work, needed to be refined based on evidence from a pilot trial^8^ to meet the diverse needs of patients in prison and to develop an implementation strategy. CBSP is a multi-component psychological intervention and when delivered by healthcare professionals in a prison environment must consider prison-related complexities.^9–12^ Consequently, the development of a logic model was necessary to delineate how the intervention was perceived to bring about its outcomes and thus support the production of an implementation strategy. This paper sets out the approach used to develop the PROSPECT logic model and the resultant initial understanding of the model's components. We aimed to develop a real-world logic model,^5^ as the PROSPECT RCT requires consideration of (a) the interplay between different model factors (e.g. how different parts of the CBSP intervention are delivered and facilitated, and by whom) and (b) how local context might affect implementation (i.e. any specific adaptations needed to successfully deliver CBSP in the prison environment and across different prisons).

## Method

### Design

**Fig. 1:**
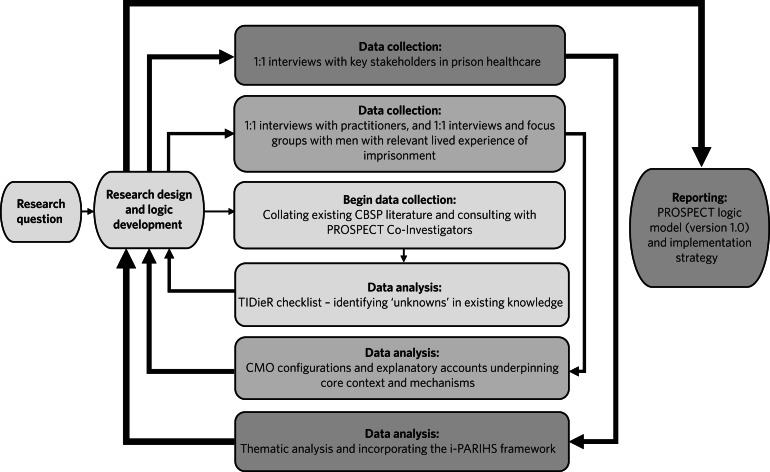
Iterative process of data collection and analysis for developing the PROSPECT logic model (adapted from Busetto et al, 2020).^13^ The process for data collection and analysis begins in the centre of the diagram and works outwards following the connecting arrows. CBSP, cognitive–behavioural suicide prevention; TIDieR, Template for Intervention Description and Replication checklist; CMO, context, mechanisms and outcomes (O); i-PARIHS, integrated-Promoting Action on Research Implementation in Health Services.

Data collection involved semi-structured qualitative one-to-one interviews scaffolded by (a) flexible and iteratively generated topic guides; (b) bespoke vignettes designed to elicit open discussion of potentially sensitive contextual factors; and (c) deliberately focused questions probing for any unknown barriers to successful implementation of the CBSP intervention in prison. Purposive sampling was used to identify and recruit participants (*n* = 28) to take part in interviews that took place over a 12-month period from November 2020 to October 2021. Inclusion criteria for participation in each stage of data collection are given in the respective sections below. As depicted in Fig. 1, data collection and analyses followed an iterative approach in which data collection tools were refined and theory developed simultaneously as the study progressed.^13,14^

As advocated by the approach of Mills and colleagues,^4,5^ features of implementation science, specifically the ‘integrated-Promoting Action on Research Implementation in Health Services’ (i-PARIHS) framework, were incorporated into the study design to develop a real-world logic model for PROSPECT, aiming to illustrate the relationships between activities and outcomes, and to consider the context where the intervention would be implemented. The i-PARIHS framework proposes that successful implementation requires consideration of four interrelated factors – (a) facilitatory components, (b) types of innovation, (c) characteristics of recipients and (d) local context^15^ – and it has proven valuable when applied to multi-site implementation projects.^16^ The i-PARIHS constructs were applied in the development of the PROSPECT logic model to optimise the subsequent process evaluation that will run concurrently with the RCT of CBSP in prison; incorporating i-PARIHS as this stage meant that problems could be tracked back and framed in a consistent way.

### Ethics

For interviews with practitioners and patients with relevant lived experience, ethical approval was obtained from the University of Manchester Research Ethics Committee (reference: 2020-9443-16034 and 2020-10252-16604). Ethical approval was obtained from the Health Research Authority and Health and Care Research Wales for interviews with prison healthcare professionals (reference: 20/SS/0021). Written or verbal informed consent was obtained from all participants. Interview recordings were transcribed verbatim and pseudo-anonymised.

### Stage 1: establishing a starting point logic

As a first step, relevant literature^8,17,18^ and descriptions of CBSP used in previous trials^7,19^ were reviewed to identify any ‘unknowns’ about implementing the CBSP intervention in prison settings. The research team's initial perceptions of their knowledge of the underlying mechanisms of the intervention, based on their clinical, research or lived experience and wider reading, were recorded, along with any prejudices and biases that could be identified. These consultations took the form of individual interviews conducted either in person or over the telephone. The research team comprised PROSPECT co-investigators with distinct experiences as clinical psychologists (*n* = 2) and lived experience of prison (*n* = 1). The chief investigator of PROSPECT (D.P.) was interviewed at three time points across the 12-month period of data collection to ensure that the developing logic was representative of their understanding. The Template for Intervention Description and Replication (TIDieR) checklist^20^ was used to extract prior knowledge about the CBSP intervention from both the existing literature and co-investigator interviews. Gaps arising from the application of the TIDieR checklist formed ‘unknowns’ and were developed into questions for subsequent interviews.

### Stage 2: including the perspective of ‘experts by experience’

In this next stage, clinical and forensic practitioners (*n* = 5) who were independent of PROSPECT but who had experience of delivering psychological interventions to similar patient groups at risk of suicide and self-harm in prisons, forensic settings and the community were consulted to understand how psychological interventions were used in these environments from their perspectives. Participants were purposively sampled via the research team's professional network. Individual interviews took place via a phone call (or via teleconferencing software) at a time convenient for the participant, and lasted approximately 45–60 min. Interviews were semi-structured and guided by a topic guide probing ‘unknowns’ that had been identified from the TIDieR checklist in stage 1. These probes were tailored to the interviewee. For example, practitioners with experience of working in secure settings provided further information on the practical aspects of delivering similar types of intervention – such as how practitioners had previously integrated clinical/forensic psychology programmes into the prison regime.

In addition to collating practitioner perspectives, interviews were also conducted with men with wide-ranging personal (lived) experiences of suicidal thoughts and/or behaviours during a period of imprisonment (*n* = 12). These interviews lasted around 60 min and aimed to understand further ‘unknowns’, specifically the perceived barriers and facilitators to engaging with the CBSP intervention in a male prison. At the time of data collection these 12 participants were residing in the community and were recruited via third-sector organisations.

### Stage 3: identifying core context and mechanisms of implementation

The aim at this stage was to understand the core context and mechanisms for implementing the CBSP intervention in prison settings. Framework analysis was used to analyse all data from stages 1 and 2 (literature extracts and interview transcripts). Data were coded for instances of related context (C), mechanisms (M) and outcomes (O), working backwards from outcomes.^21^ Data were sifted, charted and sorted, whereby the C, M and O categories were used for each column of the framework matrix and participants were assigned a separate row and grouped as either (a) existing literature, (b) PROSPECT co-investigators, (c) independent practitioner ‘experts by experience’ or (d) patient ‘experts by experience’. Separating the data in this way highlighted both convergence and divergence in the data. Any remaining unknowns or conflicting evidence were marked to be explored more deeply in the subsequent interviews and in the process evaluation of the RCT.

Where possible, expressed explanatory accounts from the CMO configurations were written in the form of ‘if … then’ statements that specified linked context, and/or mechanism and/or outcomes(s) – for example ‘If we do [MECHANISM] in [CONTEXT], then we achieve [OUTCOME]’.^22^ However, a decision was made not to limit accounts to only those expressed in this way, recognising that partial accounts were also informative at this stage – for example ‘If [CONTEXT], then [OUTCOME]’ or ‘If [MECHANISM], then [OUTCOME]’. The explanatory accounts were expressed and documented in a ‘narrative’, representing a coherent linear description of what was expected at each stage of implementation.

### Stage 4: incorporating the i-PARIHS framework

Once the core context and mechanisms for implementing the CBSP intervention in prison settings had been identified through CMO analysis, the next step was to understand how local context could affect implementation. In preparation for the next stage of data collection (stage 5), the i-PARIHS framework^15^ was introduced.

Applying i-PARIHS terminology to PROSPECT, the innovation is the CBSP psychological intervention and the training and supervision package for CBSP practitioners who deliver the therapy. Facilitation of CBSP is provided by practitioners who are clinical or forensic psychologists or cognitive–behavioural therapists.^7^ These trained practitioners might be novice facilitators (if new to the role), more experienced or even expert facilitators. The expert facilitator is responsible for training and supervising the more novice facilitators who deliver the CBSP intervention as part of the RCT. For PROSPECT the expert facilitator was the chief investigator (D.P.), who has extensive experience of delivering CBSP in prisons and developing CBSP with diverse patient groups (e.g. psychiatric in-patients, people in the community with non-affective psychosis), supervising therapists in delivering CBSP across different settings (e.g. prison, hospital, community) and working with associated National Health Service (NHS) mental health teams.

There are three categories of ‘recipient’ in PROSPECT. First, the men in prison who are perceived to be at risk of death by suicide and who participate in the CBSP programme. Second, novice facilitators delivering the CBSP programme who receive the CBSP training and supervision package. Third, the prison staff, who are considered recipients because they will affect, and be affected by, the implementation of the CBSP intervention.

The context in which PROSPECT is situated is the prison environment, including the prison culture, staff attitudes and organisational structures and priorities. PROSPECT is a multi-site trial, so it was important to consider how the local environment might differ across locations. Individual characteristics, such as prisoners’ previous experience of therapy and PROSPECT practitioners’ experience of working with people who are suicidal and/or had been in the criminal justice system, were important contextual factors that needed to be considered.

### Stage 5: developing context-sensitive implementation strategies

Four prisons in the North of England were selected to host PROSPECT and evaluate the CBSP intervention within an RCT. At this stage, we wanted to assess key stakeholders’ views on how the above i-PARIHS factors might affect, or be affected by, implementation. The aim was to investigate how these factors might be customised in different contexts, i.e. each prison site, and what each site would need for successful implementation. Often with implementation research, this is achieved by forging consensus among stakeholders.^23–25^ However, Mills and colleagues^4^ suggest that this is not appropriate for complex interventions that need adaptations to context. It was clear from the outset that PROSPECT would require localised customisation to support implementation.

For this stage of data collection, which comprised qualitative one-to-one interviews, participants were targeted who were NHS healthcare staff with experience of working with suicidal prisoners and who had supported a suicidal prisoner in the past 6 months in one of the four host prisons. Potential NHS staff participants were identified by an appropriate service manager, who sent an email on behalf of the research team to relevant members of their team. The email contained an introductory letter and a copy of the participant information sheet. Individual interviews took place via a phone call, or via teleconferencing software, at a time convenient for the participants (*n* = 5). Interviews were semi-structured, based on a topic guide comprising any outstanding ‘unknowns’ or conflicting evidence, and lasted approximately 60 min.

Using the narratives that were created following the CMO framework analysis (stage 3), a series of vignettes were designed to investigate the relationships between core underpinning mechanisms and contextual factors. Vignettes were used to provide a common context around which discussions might be shaped in the interviews with key stakeholders, reducing the need to rely on a personal frame of reference and allowing stakeholders to talk openly and without judgement. The aim was to use the vignettes to guide participants through the current understanding of how the CBSP intervention could bring about outcomes and be successfully implemented in the local prison environment.

An example vignette and related question are as follows:

‘When delivering the CBSP intervention to men in prison, practitioners will require access to a private location that can be made available for one-to-one therapy sessions to ensure confidentiality and promote engagement. It is likely that the practitioner will need access to a variety of intervention rooms throughout the prison site in order to provide easy access to the CBSP intervention for all eligible participants. Decisions on which intervention rooms can be made available to the PROSPECT practitioners should be made in collaboration with host prison staff, with PROSPECT practitioners adhering to any existing room booking procedures. Locations of intervention rooms may include interview rooms on residential wings, clinic rooms in the healthcare centre, office spaces in workshops, or interview rooms within the psychology / programmes centre.

What spaces are available for one-to-one therapy sessions and how would PROSPECT practitioners go about scheduling access to these rooms in your prison?’

This exercise was used to present the core components of implementation to identify stages of the process and ‘walk through’ the process of implementation with stakeholder participants, considering the following questions: What assumptions have we made? What have we missed? Is there anything we have not accounted for? These methods have been found to be helpful when evaluating implementation strategies in healthcare as they help ensure that procedures and protocols can be followed successfully by users in specific healthcare contexts.^26^ The aim here was to achieve context-sensitive implementation strategies, rather than consensus on context or methods (as these might be site-specific).

Researchers (R.C. and D.H.) independently analysed each transcript from the five key stakeholder interviews, following the approach to reflexive thematic analysis.^27^ Data were first analysed inductively following the thematic analysis method so that the analysis was not constricted by the i-PARIHS framework, and the codes were generated from the data using a bottom-up rather than top-down approach. Once data had been analysed in this way, the i-PARIHS framework was ‘layered’ over the top. Data-driven codes were compared with the i-PARIHS constructs and matched so that there was information for all the i-PARIHS constructs: (a) facilitatory components, (b) types of innovation, (c) characteristics of recipients and (d) local context. The data corpus was examined for instances of consensus on feasibility of implementation and also divergence regarding site-specific details on how to approach implementation. These data were then included to develop the PROSPECT logic model and programme theories to support the production of an implementation strategy. Each programme theory was written using data collected from various stakeholders throughout the study, and includes an overarching theory of change and more detailed theory of action^28^ produced by collating ‘if … then’ statements that have been refined throughout the iterative process of data collection and analysis.

## Results

### The PROSPECT logic model

**Fig. 2:**
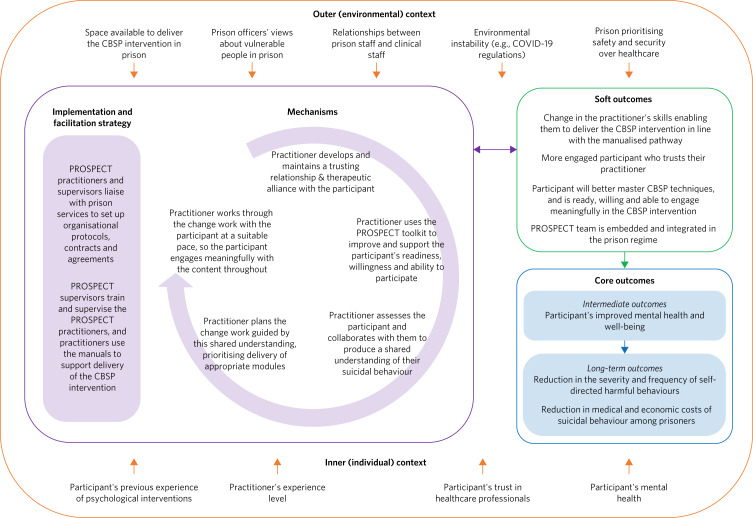
PROSPECT logic model, version 1.0. CBSP, cognitive–behavioural suicide prevention.

The PROSPECT logic model (version 1.0) (Fig. 2) and five programme theories were developed following the principles of a real-world logic model^5^ and consideration of the i-PARIHS framework.^15^ Together these describe the causal pathway of the CBSP intervention and the interplay with implementation strategies.

Context includes both inner (individual) and outer (environmental) factors at the top and bottom of the logic model; the arrows going into the centre of the model from the top and bottom in Fig. 2 represent how these contextual factors must be considered throughout implementation. Key features of the implementation and facilitation strategy are included in the left-side shaded rectangle, and the circular arrow illustrates the ordering of key mechanisms for facilitating delivery of the CBSP intervention. Soft outcomes are included in the top right-side box and core outcomes are included in the shaded boxes below. The bi-directional arrow connecting the box containing ‘Implementation and facilitation strategy' and ‘Mechanisms', and the soft outcomes shows the bidirectional relationship between these model components – i.e. activating the mechanism(s) will lead to a change in the soft outcome(s), and a change in the soft outcome(s) can affect implementation and facilitation. For example, if a more novice practitioner is supervised regularly and uses the CBSP manuals to inform intervention delivery, then there will be a change in the practitioner's skills, enabling them to deliver the CBSP intervention in line with the manualised pathway. This soft outcome will then affect the way in which they approach the intervention delivery with the participant. The arrows connecting the soft and core outcomes illustrate the relationship between outcomes – i.e. a change in soft outcomes should lead to a change in intermediate core outcomes, which should lead to a change in long-term outcomes. Constructs in the i-PARIHS framework (the innovation, context, facilitators and recipients) are embedded throughout the PROSPECT logic model.

**Fig. 3:**
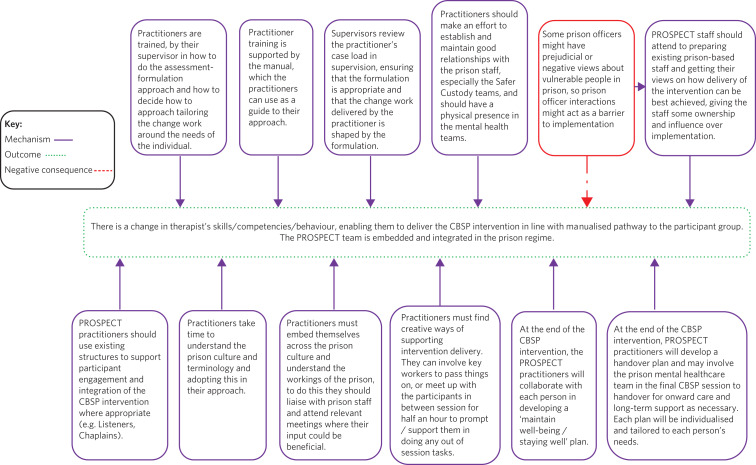
Model of the core mechanisms for Programme Theory 5: practitioner training, integrating PROSPECT and onward care (pre-, during and post-intervention). CBSP, cognitive–behavioural suicide prevention.

The different components in the larger left-side box (the ‘Implementation and facilitation strategy' and the ‘Mechanisms' for intervention delivery) provide brief summaries of programme theories that ‘sit behind’ the logic model. These provide further detail explaining the various activities and mechanisms that are required to achieve the outcomes. Drawing on the work of Brand and colleagues,^14^ each programme theory also has a diagram presenting the relationships between specific activities and the intermediate outcomes. These offer more detailed models (see Fig. 3 for an example) showing the particular mechanisms required to achieve soft outcomes for each programme theory. Broken arrows in these supplementary models indicate negative consequences that could affect the achievement of the respective intermediate outcomes. It is anticipated that if the various mechanisms that are described in the programme theories are activated and achieve the soft outcomes (included in top right-side boxes in Fig. 2), then this will lead to a change in intermediate outcomes (secondary outcome measures for the PROSPECT RCT relating to mental health and well-being) and long-term (primary) outcomes that are highlighted in shaded boxes under ‘Core outcomes' in the logic model in Fig. 2.

Programme theory 5 (PT5) is included here as an example (see Fig. 3 for the detailed model for this programme theory) and describes the core mechanisms for implementation that will develop practitioners’ skills and help integrate the CBSP intervention in the prison environment.

For each mechanism in PT5 (see the outside ‘Mechanism’ boxes connecting inward to the dotted ‘outcomes’ box in Fig. 3) there are context-sensitive factors that need to be addressed, as included in the logic model in Fig. 2. For example, when training and supervising the PROSPECT practitioners, the supervisor will need to consider the practitioners’ past work experiences and their professional background. The existing structures to support participant engagement and integration of the CBSP intervention will also be context-specific and will vary depending on which prison the PROSPECT practitioners are working in.

The five programme theories and detailed models are available in Supplementary Material 1, available at https://doi.org/10.1192/bjb.2024.22.

### Context-sensitive implementation strategy

Stakeholder interviews in the final stages of data collection provided site-specific information, including convergence and divergence. This helped generate the underlying mechanisms for each programme theory, and consideration of how these factors could differ and how implementation could be adapted to different contexts/prisons. The PROSPECT logic model (version 1.0), programme theories and detailed models supported the development of the PROSPECT implementation strategy that uses this information to guide the facilitators and recipients in the PROSPECT RCT. The implementation strategy includes the following sections: (a) information about the CBSP intervention; (b) information for prison governors and healthcare providers and commissioners; (c) information for supervisors overseeing PROSPECT; and (d) information for PROSPECT practitioners. The implementation strategy also includes site-specific sections that were produced for implementation in each of the four prisons in the RCT. These comprise key information about the i-PARIHS factors that needed to be considered and/or adapted in the programme theories for successful implementation in each prison. These include: prisoner population (recipients: e.g. the size and profile of the prisoner population); PROSPECT staff i.e. practitioners and supervisors (facilitators: e.g. what spaces are available for facilitation?); local environment (context: e.g. what does the current prison mental healthcare model look like and what are staff attitudes to suicidal prisoners?); and the CBSP intervention (innovation: e.g. what other psychological interventions are available and how does CBSP compare?).

## Discussion

The PROSPECT logic model (version 1.0) was developed to articulate how the CBSP intervention brings about its outcomes in the prison environment and to support the implementation of the CBSP intervention with male participants in prison. Drawing on the work of Mills and colleagues^4,5^ and also Brand and colleagues,^14^ a logic model and related programme theories illustrating and describing the relationships between activities (mechanisms) and outcomes were generated, while also considering how context can affect implementation. For PROSPECT, the CBSP intervention is being implemented as part of a wider programme of work, specifically in the treatment arm of an RCT whereby half of the participants enrolled into the trial will be offered the intervention over a period of 6 months. The PROSPECT programme theories are now being utilised in the PROSPECT RCT and concurrent process evaluation. The outcome of the RCT will tell us ‘what’ – i.e. is the CBSP intervention clinically effective and cost-effective when implemented in prison? – whereas the process evaluation will tell us ‘how’ and ‘why’, and findings will be considered to develop and refine the PROSPECT logic model for further implementation, as appropriate.

### Strengths and limitations of the PROSPECT logic model

Logic models are often used in healthcare research (for examples, see^4,29–31^). Nevertheless, critiques have noted some key limitations, namely, that they can overlook the complexity underlying interventions, limit exploration of adaptations to enhance contextual compatibility or disregard any undesirable outcomes.^32^ By developing a real-world logic model^5^ we aimed to navigate these potential limitations. Specifically, the PROSPECT logic model and programme theories consider context throughout implementation, and interviews with key stakeholders at different prison sites allowed for local contextual factors and adaptations to be included. This helped shape context-sensitive implementation strategies, and potential undesirable outcomes that could affect implementation are included in the programme theories and respective diagrams.

That is not to say that the current version is without flaws. Some features of the logic model will require attention. As described earlier, the expert facilitator of CBSP for PROSPECT is the chief investigator (D.P.); logic model development in the process evaluation will need to account for not having this role for wider implementation outside of the trial, among other factors, as D.P. would not have capacity to train and supervise all CBSP practitioners if the intervention were successful and rolled out across the prison estate. Likewise, outside of the PROSPECT RCT, trained psychologists would need to be employed to deliver the CBSP intervention in prison. In an environment where the few trained psychologists working in prison are in high demand, these roles would require funding and resourcing – contextual challenges that require further investigation in the PROSPECT process evaluation.

An additional limitation of the current model is that, owing to restrictions surrounding research activity during the COVID-19 pandemic,^33^ we were not able to interview staff employed by His Majesty's Prison and Probation Service, so these perspectives are not included in the current version of the logic model. Prison staff participants will be approached for interviews in the process evaluation of PROSPECT and their perspectives will be included in the next iteration of the model and developing programme theories. By using the PROSPECT logic model to inform implementation during the trial we can test and evaluate our current understanding of how the CBSP intervention should bring about its outcomes. This will be done through rigorous qualitative analysis in the process evaluation. The process evaluation method includes further investigation of the barriers and facilitators to implementation, and has the benefit of patient and public involvement (both staff and prisoners) actively experiencing implementation of the CBSP intervention during the RCT, and contributing to the logic model development.

### Further development of the PROSPECT logic model

Novel findings from this study highlight the value of developing a real-world logic model that considers both the causal relationships between model factors *and* the context of the intervention^4,5^ as a crucial initial step in supporting implementation of any complex psychological intervention trial. Recent advances in implementation science, alongside the RWLM, have introduced the implementation research logic model (IRLM).^2^ Rather than applying implementation framework(s) to the logic of the intervention itself (as we did with i-PARIHS for PROSPECT), the IRLM integrates implementation science into the core logic model, with the aim of improving the rigour and reproducibility of implementation research, and this has been shown to provide structure when developing causal pathways and mechanisms.^3^ Developments in the PROSPECT logic model could consider this approach, drawing on learnings from producing an RWLM in line with guidance from Mills and colleagues^5^ and considering the most effective way of illustrating and guiding implementation. Further developments should include stakeholders in the final developments of the model, ensuring that the programme theories for implementing the CBSP intervention in prison, and specifically the visualisation of implementation (PROSPECT logic model), could support implementation beyond the research trial.^34^

## Supporting information

Crook et al. supplementary material Crook et al. supplementary material

## Data Availability

The data that support the findings of this study are available from the corresponding author, D.P., on reasonable request.

## About the authors

**Rebecca Crook** is a research associate in the School of Health Sciences, Faculty of Biology, Medicine and Health, University of Manchester, Manchester Academic Health Science Centre, Manchester, UK and in the Suicide Risk and Safety Unit, Greater Manchester NHS Foundation Trust, Manchester, UK. **Charlotte Lennox** is a senior lecturer in psychology and mental health in the School of Health Sciences, Faculty of Biology, Medicine and Health, University of Manchester, Manchester Academic Health Science Centre, Manchester, UK. **Yvonne Awenat** is a senior clinical research fellow in the School of Health Sciences, Faculty of Biology, Medicine and Health, University of Manchester, Manchester Academic Health Science Centre, Manchester, UK and a director of the Suicide Risk and Safety Unit, Greater Manchester NHS Foundation Trust, Manchester, UK. **Dawn Edge** is Professor of Mental Health and Inclusivity in the School of Health Sciences, Faculty of Biology, Medicine and Health, University of Manchester, Manchester Academic Health Science Centre, Manchester, UK. **Sarah Knowles** is a research fellow in knowledge mobilisation in the Centre for Reviews and Dissemination, University of York, York, UK. **David Honeywell** is a lecturer in criminology in the School of Criminal Justice, Arden University, Coventry, UK. **Patricia Gooding** is a senior lecturer in psychology in the School of Health Sciences, Faculty of Biology, Medicine and Health, University of Manchester, Manchester Academic Health Science Centre, Manchester, UK and a director of the Suicide Risk and Safety Unit, Greater Manchester NHS Foundation Trust, Manchester, UK. **Gillian Haddock** is Professor of Clinical Psychology in the School of Health Sciences, Faculty of Biology, Medicine and Health, University of Manchester, Manchester Academic Health Science Centre, Manchester, UK and a director of the Suicide Risk and Safety Unit, Greater Manchester NHS Foundation Trust, Manchester, UK. **Helen Brooks** is a senior lecturer in nursing and midwifery in the School of Nursing, Midwifery and Social Work, Faculty of Biology, Medicine and Health, University of Manchester, Manchester, UK. **Daniel Pratt** is a senior lecturer in clinical psychology in the School of Health Sciences, Faculty of Biology, Medicine and Health, University of Manchester, Manchester Academic Health Science Centre, Manchester, UK and director of the Suicide Risk and Safety Unit, Greater Manchester NHS Foundation Trust, Manchester, UK.
