# Supplementary material for: Development of the Prevention of Suicide Behaviour in Prisons: Enhancing Access to Therapy (PROSPECT) logic model and implementation strategies
Source: BJPsych Bull. 2024 Oct;48(5):285–93. doi: 10.1192/bjb.2024.22 (PMC11649361; doi:10.1192/bjb.2024.22)
Supplement: Crook et al. supplementary material [file S2056469424000226sup001.docx]

## Supplementary material 1: PROSPECT programme theories

**PT1 – Trust, willingness and engagement (throughout the CBSP intervention)**

*Theory of change:* If the practitioner develops a trusting relationship and therapeutic alliance with the individual, and the individual is willing to participate in the intervention and engages with the practitioner and the intervention content, then this will lead to an overall improvement in [measurable outcomes] and then a reduction in [long-term outcome/s].

*Theory of action:* Trust is a key factor in ensuring that men in prison will engage meaningfully in the CBSP intervention, and the most important way that the PROSPECT practitioner can support engagement is by developing a trusting relationship and therapeutic alliance. Men in prison are likely to mistrust authority figures; PROSPECT practitioners build trust by showing sincere compassion for, and interest in, the individual, and working in a flexible, person-centred way that shows the individual that the PROSPECT practitioner is genuine. There’s also importance attached to the practitioner being regularly and predictably available. So, the practitioner aims to avoid missing pre-arranged sessions, and any sessions that must be missed by the practitioner are communicated to the individual beforehand, wherever possible. This idea builds upon the notion of a ‘Secure Base’ (from attachment theory), whereby a secure base is built through the predictable availability and reliability of the practitioner’s offer to meet with the participant. When the participant develops a confidence in the practitioner’s availability and reliability, then they can begin to feel safe in the knowledge that they can return to their secure base (i.e., the practitioner) for help if needed. This is akin to a child developing trust in their caregiver (e.g., parent), whereby once this safe and secure base and trust has been established the child can then become confident to explore the world around them. And so, establishing trust with their practitioner may help to support the participant to become confident in their exploration of new ways of coping with suicidal thoughts and behaviours.

If the individual has had earlier negative experiences of support and / or the PROSPECT practitioner is seen as part of the ‘establishment’ then this could create a barrier to building trust; hence, the PROSPECT practitioner should have an informal appearance that is distinguishable from other staff working in the prison – i.e., own clothes / informal dress. They should discuss any concerns the individual might have and listen to them and their experiences non-judgementally, then the individual will begin to trust their practitioner. To support this, PROSPECT practitioners will be trained how to approach initial interactions with men in prison, including appropriate verbal and non-verbal language, by someone with lived experience of being in prison. As trust building can take time, it is important that each individual meets with the same practitioner throughout their involvement in the intervention, and that these meetings are regularly and predictably available, where possible.

One important way in which a PROSPECT practitioner can encourage willingness to engage in the CBSP intervention is to provide clear and concise information about the CBSP intervention to men in prison who have consented to take part. All important information is available in the PROSPECT toolkit, which has been co-designed with individuals with lived experience, ensuring that the content and presentation is appropriate. The practitioner provides the individual with information about the intervention and explains this to them. This will help the individual to feel more informed and familiar with a talking therapy intervention in general, and then they are more willing to engage with the intervention. A lack of clarity and information could make them unsure and not want to engage; if they are not informed about what to expect from the CBSP intervention or from their practitioner, then they might not see the credibility of the intervention and be mistrusting of the practitioner. Having little or no experience of participating in talking therapy might mean that the individual is apprehensive or unwilling to engage in the intervention, so when providing information, and discussing the intervention content, this is also an opportunity for the practitioner to work collaboratively with the individual and mutually agree clear boundaries and expectations held by the practitioner and the individual. This might include discussing the time and commitment needed to successfully engage in the intervention, and / or what topics the individual is / isn’t currently comfortable discussing. The ‘talking about how you feel’ section of the PROSPECT toolkit can support the practitioner’s approach to this conversation. This will help the individual to see credibility in how the intervention could benefit them and they will gain a greater appreciation of what is expected from them, and what to expect from the practitioner. This will help strengthen the individual’s willingness to engage in the intervention and serve to strengthen their therapeutic alliance with the practitioner prior to commencing with the ‘change work’ stage of the intervention.

Men in prison, and specifically individuals approached to participate in the CBSP intervention, which may involve talking about sensitive topics such as suicide, need to be clear about confidentiality and privacy. The PROSPECT practitioner will provide a suitable location in the prison to have 1:1 sessions, which is private and comfortable, and will also be clear with the individual about limits of confidentiality. This privacy, honesty and transparency is crucial to building trust and supporting engagement.

More engaged participant who trusts the practitioner and is willing to participate in the CBSP intervention

A lack of clarity or lack of accessible information could make the participant unsure and not want to engage; they might not see the credibility of the intervention and be mistrusting of the practitioner.

Practitioner discusses any concerns the individual might have and listens to him and his experiences non-judgementally.

The practitioner works collaboratively with the individual and they mutually agree clear boundaries and expectations held by the practitioner and the individual. They could use the ‘talking about how you feel’ section of the PROSPECT toolkit here.

The participant meets with the same practitioner throughout their involvement in the intervention.

Practitioners are trained how to approach initial interactions with men in prison, including appropriate verbal and non-verbal language, by someone with lived experience of being in prison.

Practitioners build trust by showing sincere compassion for, and interest in, the individual, and working in a flexible, person-centred way that shows the individual that the PROSPECT practitioner is genuine.

PROSPECT practitioner should have an informal appearance that is distinguishable from other staff working in the prison

The practitioner provides the individual with clear and concise information about the CBSP intervention and explains this to them, developing an informed understanding of what to expect from the CBSP intervention.

The practitioner is regularly predictably, and reliably available and aims to avoid missing pre-arranged sessions.

The PROSPECT practitioner provides a suitable location in the prison to have 1:1 sessions, which is private and comfortable, and is clear with the individual about limits of confidentiality.

**PT1 – Trust, willingness and engagement (throughout the CBSP intervention)**

**PT2 – Readiness and ability (preparatory stage)**

*Theory of change:* In order for an individual to move on from the preparatory stage of the CBSP intervention, they must be ready, willing and able; this readiness, willingness and ability (drawing from the Motivational Interviewing literature e.g. Britton *et al.*, 2008) must be assessed by the practitioner, and where necessary, resources from the PROSPECT toolkit can be utilised to improve and support readiness, willingness and ability. **Only** when the individual is ready, willing, and able to move on the change work will they have more successful engagement with the intervention content, then this will lead to an overall improvement in [measurable outcomes] and then a reduction in [long-term outcome/s].

*Theory of action:* The PROSPECT toolkit is the way through which the CBSP intervention enables a number of mechanisms to support individuals to work towards being more ready, willing and able to engage with the ‘change work’ stage of the CBSP intervention.

An important mechanism to ensure that individuals are ready to engage with the change work stage of the CBSP intervention is to ensure that they are open to talking about their feelings and emotions. To do this, resources are included in the toolkit to support PROSPECT practitioners in broaching conversations about feelings and suicide with participants who might be resistant or find it difficult to do so. Practitioners will be trained to understand when and why an individual might be resistant, or find it challenging, to talk about these topics, so that they will know when it is appropriate to utilise these resources. If used at the right time and in the right way, these resources, and the practitioner’s approach, will help to make the individual feel at ease and comfortable. They will then be more likely to open up and be honest with the practitioner, and then ready to move on to the change work stage. This need for openness is important but barriers to ‘being open to talk about my feelings’ may come from an unwillingness and / or an inability to do so. The resources in the toolkit have been developed to support the practitioner to address any potential inability held by the individual, but these resources may be less relevant or helpful for the practitioner trying to address an unwillingness to talk about feelings. When working with unwillingness, the practitioner would also be guided more towards adopting a Motivational Interviewing approach to their conversations with the clients.

To support individuals to feel more able to talk about their emotions and feelings, other resources that are based on existing psycho-education activities are included in the toolkit. These resources are designed to explore different emotions and how these can be linked to associated thoughts, feelings and behaviours. The practitioners will be trained by their supervisor to assess when to utilise this section of the toolkit, and how each resource should be used. The aim is to provide a space for shared learning about emotions, meaning that the practitioner can learn more about what challenges the individual might have when asked to express their emotions and discuss their feelings, and then use the resources in the toolkit to develop the individual’s ability to talk about their emotions and feelings. Men in prison will have varying degrees of literacy and emotional literacy. The practitioner will know how to assess this, as directed by the PROSPECT training and manual, and will utilise the toolkit using a person-centred approach, making a collaborative decision with the individual about which resources might be helpful to them. Appropriate use of the resources will mean that individuals will have a better understanding of emotions and improved emotional literacy, but if they are presented at the wrong time or at a pace that is too fast or too slow, this may encourage disengagement and won’t work.

PROSPECT practitioners that are experienced in working with CBT-based interventions or similar talking therapies might consider using alternative resources or techniques (such as motivational interviewing) to promote readiness, willingness, and ability of individuals that they meet with when delivering the CBSP intervention. If they wish to utilise other resources that aren’t already included in the toolkit, this should be discussed with their supervisor and any changes to the toolkit must be manualised across all practitioners to ensure consistency of approach.

While the PROSPECT toolkit is mostly targeted for use in the engagement phase of the initial preparatory stage, it can be used and re-used throughout the intervention. If the practitioner finds an individual to be struggling with some of the change work modules, the practitioner should consider utilising some of the toolkit resources to help support the individual in building up their ability to meaningfully engage with and access the change work content.

More engaged participant who is ready and able to participate in the CBSP intervention

If Toolkit resources are presented at the wrong time or at a pace that is too fast or too slow, this may encourage disengagement and won’t work.

Experienced practitioners use alternative resources or techniques to promote the participant’s readiness and ability. Any changes are discussed with the supervisor and manualised.

If appropriate, the practitioner utilises the Toolkit resources throughout the CBSP intervention, if/when they feel that it would support the participant’s readiness and ability to engage.

The practitioner learns more about what challenges the individual might have when asked to express their emotions and discuss their feelings.

Practitioner utilises the ‘talking about how you feel’ section in the Toolkit when broaching conversations about feelings and suicide with participants who might be resistant or find it difficult to do so.

Practitioners are trained to understand when and why an individual might be resistant, or find it challenging, to talk about their feelings, so that they will know when it is appropriate to utilise these resources.

When working with unwillingness, the practitioner would also be guided more towards adopting a Motivational Interviewing approach to their conversations with the clients.

Practitioner utilises appropriate resources from the Toolkit at a suitable pace and when they are most useful to the participant.

The practitioner will know how to assess the participant’s ability in talking about their emotions, as directed by the PROSPECT training and manual.

The practitioner will utilise the toolkit using a person-centred approach, making a collaborative decision with the individual about which resources might be helpful to them.

**PT2 – Readiness and ability (preparatory stage)**

**PT3 – Assessment and formulation (preparatory stage)**

*Theory of change:* During the process of engaging an individual, establishing a therapeutic alliance, and ensuring that they are willing, ready, and able to progress from the preparatory stage of the intervention on to the change work stage, the practitioner will make an assessment of the person. This assessment will identify patterns of behaviours and they will collaboratively build up a formulation (a shared understanding) of the individual’s suicidal behaviour. The practitioner will plan the change work stage of the intervention, guided by this formulation, prioritising delivery of appropriate modules. This means that the change work is tailored to the individual’s formulation, which will lead to an overall improvement in [measurable outcomes] and then a reduction in [long-term outcome/s].

*Theory of action:* A vital part of the CBSP intervention is the preparatory stage, which comprises an ‘engagement phase’ (addressed in Programme Theories 1 and 2) and an ‘assessment phase’. The assessment phase aims to 1) identify how suicidal thoughts and behaviours fit into concerns; 2) establish a shared understanding (formulation); and 3) strengthen a commitment to change and eliciting goals for therapy. Developing a shared understanding is a key way in which the PROSPECT practitioner enables the intervention participant to feel willing, ready and able to move into ‘change work’ and subsequently work through the content of the CBSP intervention. During the assessment phase the practitioner will assess the individual’s thoughts and behaviours around suicide, and together they will collaboratively decide how the CBSP intervention can be tailored around the individual’s needs. An output of this phase is a collaboratively developed formulation of their experience(s) of suicide ideation and behaviour. It is this formulation that influences the planning of the delivery of the ‘change work’.

Working together with someone to build an understanding of their unique situation involves both gathering background information and identifying the person’s needs and goals, and making links between a person’s emotions, thinking, and behaviour. The process of developing a shared understanding provides an early and ongoing opportunity for the individual to name and rename the problem, shift perspectives from deficits to strengths and create opportunities for them to have a voice in shaping the method for solving problems or meeting goals they have identified. Identifying priority 'problems' that the person wants to address and reframing these problems in terms of goals and needs, is an example of how practitioners can support individuals to shift their perspectives of the 'problem', their strengths, and their potential to change. Practitioners can support someone to identify their priorities by facilitating their understanding of their own narrative / journey and their social and cultural context.

The practitioner speaks to the individual about recent, or more salient, episodes that have triggered thoughts about suicide, on the understanding that more recent or salient memories are most accessible to the client. Recall of these episodes allows the practitioner and client to ‘unpick’ what the client’s thoughts, feelings and behavioural responses were at the time of the suicidal crisis, in the moments preceding the crisis (to identify potential antecedents) and in the moments following the crisis (to identify potential consequences). The PROSPECT toolkit and staying well plan offer resources to support this development of timelines of previous suicidal crises. Drawing upon the information revealed through this review of previous suicidal episodes, the practitioner and client can then begin to build the formulation and identify potential exits (away from suicidal thinking) to goals for the intervention work. This collaborative work can start off fairly simply and means that they can look to work on what immediate things can be helped. There could be environmental factors that contribute to an individual’s suicidal thoughts or behaviours, these need to be acknowledged and built into the formulation, this will help the formulation to offer a more accurate understanding. Then they can build on the formulation by looking back and identifying patterns of behaviour, looking at what events have previously contributed to the activation of the core suicide schema and what events can trigger, this then helps develop a long-term formulation (typically referred to as a ‘longitudinal formulation’). The ideal procedure would be to have a detailed formulation of how problems have built up over the life intervention to allow for most ideal method of delivering the change work modules, but this varies between individuals, meaning that the actual order of the change-work modules and specific content within each module delivered might differ between individuals, but the formulation-driven approach is consistent, at a principle level.

Men in prison might have varying experiences and abilities of talking about their emotions and engaging with a CBT-based talking therapy, which attaches a lot of importance to thinking. If the practitioner assesses the individual and feels that they aren’t ready and / or able to move on to the change work, then they should utilise the PROSPECT toolkit to support the person’s development until they are ready and able (see PT2). For those with less experience or find this more challenging, the aim is to: encourage them to start putting into words what they were thinking during a particular episode; encourage them to become more familiar with and socialise themselves to a psychological model and a psychological understanding of their experience – i.e. why they do what they do, according to psychological theory (specifically the SAMS model). The individual needs to be supported to better understand their thinking so that they can then understand how thinking relates to what they’re feeling, and how their thoughts and feelings influence what they then do; this cognitive model can then be applied to their suicidal thoughts and this is the beginning of a formulation of their suicidal ideation and behaviour, which subsequently informs intervention planning. When this work is complete, the individual can then collaborate with the practitioner to decide which of the five modules to work through; this is a decision made jointly by the practitioner and the individual, influenced by the formulation they’ve developed together.

There is no prescribed order in which all participants are expected to complete the change work modules, the specific order for each individual is jointly decided by the practitioner and individual. The practitioner prioritises what they expect the individual to find most amenable, easy to understand, and achievable, coupled with a consideration of what the formulation suggests would be most appropriate, but without forcing this priority and being mindful of returning to this decision-making when needed – engagement is the priority. If the individual is forced to complete a module that they don’t want to, they may drop out or become disengaged, but also, if an important module (as identified by the formulation approach) is missed that might mean that change for some outcomes aren’t as significant.

For some individuals, it may take a long time to develop a trusting relationship between the practitioner and the individual, and for them to be ready, willing, and able to engage with the formulation approach, due to individual differences and mastery of techniques. In these instances, the practitioner should take time to develop trust (see PT1) and also be open to seeing benefits in other outcomes that aren’t directly referred to in the change work, such as environmental outcomes or personal outcomes (e.g. improved sleep or personal hygiene), and consider these in the formulation, appreciating the therapeutic benefit from things that might not be listed as primary or secondary outcomes that could still be a successful outcome for that individual.

Practitioner and participant have a ‘shared understanding’ (formulation) of the participant’s suicidal thoughts and behaviours, and they have a plan for the ‘change work’ stage.

The participant can better master techniques needed to engage meaningfully with change work modules of the CBSP intervention.

Practitioner supports the individual to shift their perspectives of the 'problem', their strengths, and their potential to change by facilitating their understanding of their own narrative / journey and their social and cultural context.

The practitioner and participant ‘unpick’ what the individual’s thoughts, feelings and behavioural responses were at the time of the suicidal crisis, in the moments preceding the crisis, and also in the moments following the crisis.

The individual names and renames their problems, shifting perspectives from deficits to strengths.

The practitioner assesses the individual’s thoughts and behaviours around suicide, and together they collaboratively decide how the CBSP intervention can be tailored around the individual’s needs.

The practitioner gathers background information and identifies the participant’s needs and goals, making links between their emotions, thinking, and behaviour.

The practitioner and the participant identify priority 'problems' that the person wants to address and reframe these problems in terms of goals and needs.

The practitioner supports the individual to better understand their thinking so that they can then understand how thinking relates to what they’re feeling, and how their thoughts and feelings influence what they then do.

The participant has a voice in shaping the method for solving problems or meeting goals they have.

Practitioner takes time to develop trust (see PT1) and is open to seeing benefits in other outcomes that aren’t directly referred to in the change work, appreciating the therapeutic benefit from things that could still be a successful outcome for that individual.

If the practitioner feels that the participant isn’t ready and / or able to move on to the change work, then they should utilise the PROSPECT toolkit to support the person’s development until they are ready and able (see PT2).

The practitioner prioritises what they expect the individual to find most amenable and achievable, while considering what the formulation suggests would be most appropriate – maintaining engagement is the priority.

If the individual is forced to complete a module that they don’t want to, they may drop out or become disengaged. If an important module is missed that might mean that change for some outcomes aren’t as significant.

The practitioner speaks to the individual about recent, or more salient, episodes that have triggered thoughts about suicide.

The practitioner and participant build a detailed formulation of how problems have built up over the life intervention to allow for most ideal method of delivering the change work modules.

**PT3 – Assessment and formulation (preparatory stage)**

**PT4 – Therapist delivering ‘change work’ stage of the CBSP intervention face-to-face in prison environment (change work stage)**

*Theory of change*: The second stage of the CBSP intervention is the ‘change work’ stage, comprising the delivery of different modules of the CBSP intervention, with ordering of delivery and content of each module tailored according to the individual’s formulation. The practitioner will deliver the module content and work through the change work with the individual at a suitable pace, and ensuring that the individual is willing, ready, and able to engage with the content throughout, returning to the Toolkit resources whenever appropriate. If the relevant change work is successfully delivered and engaged with, then the individual will see an overall improvement in [measurable outcomes] and then a reduction in [long-term outcome/s].

*Theory of action*: Following the assessment and developing a working formulation of the individual’s suicidal ideation and behaviour, the change work can commence. A number of key mechanisms must be activated to enable the individual to successfully work through the change work stage of the CBSP intervention. In agreement with the individual, the practitioner will plan the change work and structure the intervention prioritising modules based on the previously agreed formulation. The practitioner will provide clear information about the modules, including aims and content, and they will use suitable language that does not position any problem within the person, but rather as something that the person is doing or is being done to them. If the practitioner fails to do this, the individual might feel as though they are being blamed for problems that they are facing, or have faced, and this could hinder subsequent engagement and success. Working on modules tailored to the individual shows them that the practitioner has understood and listened to them and gives the individual a role in decision-making in the intervention, helping them establish and work towards their own goals. This will enable the individual to engage meaningfully with the modules that are best suited to them.

The change work stage of the CBSP intervention is delivered following the preparatory stage, in five distinct modules: 1) Attention Broadening; 2) Cognitive Restructuring; 3) Problem-Solving Training; 4) Mood Management & Behavioural Activation; and 5) Improving Self-Esteem & Positive Schema. Each change work module has its own focus, and therapeutic objectives and techniques, and the ordering of the modules is subject to the individual formulation. The practitioner will deliver prioritised modules first but can deliver multiple therapeutic techniques within a given session, meaning that a more comprehensive targeting of the psychology behind the patient’s suicidal thinking is offered. An excessive number of techniques could undermine the focus of the session or confuse the individual, so practitioners are advised to limit the concurrent delivery of therapeutic techniques to no more than three techniques within a single session. It would be most typical for the practitioner to deliver one or two modules within a single session.

When delivering the Attention Broadening module, the practitioner supports the individual to use attention training techniques that help the individual learn how to become more in control of what they focus their mind on. Using these techniques will reduce the power of threat-focussed attention and other information processing biases that maintain the activation of the suicide schema. In sessions focussed on Cognitive Restructuring, the practitioner supports the individual with psychoeducation work that focuses on improving their awareness of common thinking biases. The individual will then be supported to look at their thoughts and understand more about where they have come from and about their thinking patterns. They will then begin to challenge the accuracy and usefulness of any unhelpful beliefs in order to develop more helpful, accurate thoughts and beliefs about themselves, their future and the world around them. In the Problem-Solving Training module, the practitioner supports the individual to use a structured and systematic approach and develop skills in improving solving problems, then the individual can use problem-solving techniques to inform the development of more positive appraisals of coping in future situations. For Mood Management & Behavioural Activation, if the individual is in a state of hopelessness, defeat or entrapment, and the individual is supported to timetable a greater number of activities previously found to be associated with an increased sense of pleasure and/or achievement into their daily or weekly schedule, then this ensures routine access to positive, alternative schema that would strengthen the individual’s resilience, sense of personal agency, and control over their life. To Improve Self-Esteem & Positive Schema, the practitioner supports the individual to enhance activation of more positive schemas with associated links with adaptive problem-solving responses. The individual will then adopt new and appropriate schematic beliefs about their circumstances, self, and future, which will promote self-worth and deactivate, inhibit and / or change the suicide-relate schema.

Participant remains engaged, and will better master techniques needed to engage meaningfully with change work modules that are best suited to them.

When delivering the Attention Broadening module, the practitioner supports the individual to use attention training techniques that help the individual learn how to become more in control of what they focus their mind on.

In sessions focussed on Cognitive Restructuring, the practitioner supports the individual with psychoeducation work that focuses on improving their awareness of common thinking biases.

An excessive number of techniques could undermine the focus of the session or confuse the individual, so practitioners are advised to limit the concurrent delivery of therapeutic techniques to no more than three techniques within a single session.

In agreement with the individual, the practitioner plans the change work and structures the intervention prioritising modules based on the previously agreed shared understanding (the formulation).

Practitioner provides clear information about the modules, including aims and content, and uses suitable language that does not position any problem within the person, but as something that the person is doing or is being done to them.

If the practitioner fails to do this, the individual might feel as though they are being blamed for problems that they are facing, or have faced, and this could hinder subsequent engagement and success.

For Mood Management & Behavioural Activation, the practitioner supports the individual to timetable more activities previously found to be associated with an increased sense of pleasure and/or achievement into their daily or weekly schedule.

The practitioner will deliver prioritised modules first but can deliver multiple therapeutic techniques within a given session. It would be most typical for the practitioner to deliver one or two modules within a single session.

In the Problem-Solving Training module, the practitioner supports the individual to use a structured and systematic approach and develop skills in improving solving problems.

To Improve Self-Esteem & Positive Schema, the practitioner supports the individual to enhance activation of more positive schemas with associated links with adaptive problem-solving responses.

**PT4 – Therapist delivering ‘change work’ stage of the CBSP intervention face-to-face in prison environment**

**(change work stage)**

**PT5 – Practitioner training, integrating PROSPECT, and onwards care (pre and post intervention)**

*Theory of change*: If the PROSPECT practitioners are trained by the supervisors, and the supervisors utilise the Intervention Delivery Platform (the PROSPECT manuals, the training package, and the supervision package), then the practitioners will be able to successfully deliver the CBSP intervention. If the PROSPECT team is embedded and integrated in the prison regime then this will help implement the CBSP intervention and support ongoing care for those who participate.

*Theory of action*: Practitioners will be trained, by their supervisor, in how to do the assessment-formulation approach and how to decide how to approach tailoring the change work around the needs of the individual; this training will be supported by the manual, which the practitioners can use as a guide to their approach. Supervisors will review practitioner’s case load in supervision, ensuring that the formulation is appropriate and that the change work delivered by the practitioner is shaped by the formulation. Access to this training, manual and supervision will activate a change in the practitioner’s skills, competencies, and behaviour to enable them to deliver the intervention in line with the manualised pathway with any individual.

A number of factors will support successful integration of the PROSPECT team, and the intervention, into the prison regime. Prison staff might have negative perceptions of the PROSPECT team and of the intervention, and this could be a potential barrier to successful implementation. Practitioners should make an effort to establish and maintain good relationships with the prison staff, especially the Safer Custody teams, and should have a physical presence in the mental healthcare teams. Healthcare delivery is not the prison’s primary purpose, so PROSPECT staff should do plenty of work preparing existing staff and getting their views on how delivery of the intervention can be best achieved, giving the staff some ownership and influence over the implementation of the intervention. Some prison officers might have prejudicial or negative views about vulnerable people in prison, so prison officer interactions might act as a barrier to engagement. PROSPECT practitioners should use existing structures to support engagement e.g., Listeners, Chaplains. Some participants might not be in contact with the mental healthcare team or other services, but might request access. In these instances, PROSPECT practitioners can signpost / refer if the participant wants and consents to this. Practitioners should provide clear information about this so the individual is fully informed. Linking with other services can support onward care once the CBSP intervention is completed. Integration is also supported by the practitioners taking time to understand the prison culture and terminology and adopting this in their approach. The practitioners must embed themselves across the prison culture and understand the workings of the prison, to do this they should liaise with prison staff and attend relevant meetings where their input could be beneficial, e.g., ACCT reviews of individual’s completing the CBSP intervention. Practitioners must find creative ways of supporting intervention delivery, and this will likely be different across different prison sites; delivering this type of intervention in prison is much different to the community. Practitioners can involve key workers to pass things on, or meet up with the participants in between session for half an hour to prompt / support them in doing any out of session tasks, this way the intervention is more embedded into the prison regime.

Following the CBSP intervention, which lasts six months, individuals may require onward care pathways and ongoing support. The PROSPECT practitioners will collaborate with each person in developing a ‘maintain wellbeing / staying well’ plan. They will also develop a handover plan and may involve the prison mental healthcare team in this and invite someone from the team to join in the final intervention session to handover for onward care and long-term support as necessary. Each plan will be individualised and tailored to each person’s needs. Ongoing support will help maintain any positive outcomes from the CBSP intervention.

There is a change in therapist’s skills/competencies/behaviour, enabling them to deliver the CBSP intervention in line with manualised pathway to the participant group.

The PROSPECT team is embedded and integrated in the prison regime.

PROSPECT practitioners should use existing structures to support participant engagement and integration of the CBSP intervention where appropriate (e.g. Listeners, Chaplains).

Practitioners take time to understand the prison culture and terminology and adopting this in their approach.

PROSPECT staff should do plenty of work preparing existing staff and getting their views on how delivery of the intervention can be best achieved, giving the staff some ownership and influence over the implementation of the intervention.

Practitioners are trained, by their supervisor in how to do the assessment-formulation approach and how to decide how to approach tailoring the change work around the needs of the individual.

Practitioner training is supported by the manual, which the practitioners can use as a guide to their approach.

Supervisors review the practitioner’s case load in supervision, ensuring that the formulation is appropriate and that the change work delivered by the practitioner is shaped by the formulation.

Practitioners must find creative ways of supporting intervention delivery. They can involve key workers to pass things on, or meet up with the participants in between session for half an hour to prompt / support them in doing any out of session tasks.

Practitioners should make an effort to establish and maintain good relationships with the prison staff, especially the Safer Custody teams, and should have a physical presence in the mental health teams.

Practitioners must embed themselves across the prison culture and understand the workings of the prison, to do this they should liaise with prison staff and attend relevant meetings where their input could be beneficial.

At the end of the CBSP intervention practitioners will develop a handover plan and may involve the prison mental healthcare team in this and invite someone from the team to join in the final intervention session to handover for onward care and long-term support as necessary. Each plan will be individualised and tailored to each person’s needs.

At the end of the CBSP intervention the PROSPECT practitioners will collaborate with each person in developing a ‘maintain wellbeing / staying well’ plan.

Some prison officers might have prejudicial or negative views about vulnerable people in prison, so prison officer interactions might act as a barrier to implementation.

**PT5 – Practitioner training, integrating PROSPECT, and onwards care (pre, during and post intervention)**
